# Genome-Wide Analysis of Selective Constraints on High Stability Regions of mRNA Reveals Multiple Compensatory Mutations in *Escherichia coli*

**DOI:** 10.1371/journal.pone.0073299

**Published:** 2013-09-27

**Authors:** Yuanhui Mao, Qian Li, Yinwen Zhang, Junjie Zhang, Gehong Wei, Shiheng Tao

**Affiliations:** 1 College of Life Sciences and State Key Laboratory of Crop Stress Biology in Arid Areas, Northwest A&F University, Yangling, Shaanxi, China; 2 Bioinformatics Center, Northwest A&F University, Yangling, Shaanxi, China; British Columbia Centre for Excellence in HIV/AIDS, Canada

## Abstract

Message RNA (mRNA) carries a large number of local secondary structures, with structural stability to participate in the regulations of gene expression. A worthy question is how the local structural stability is maintained under the constraint that multiple selective pressures are imposed on mRNA local regions. Here, we performed the first genome-wide study of natural selection operating on high structural stability regions (HSRs) of mRNAs in *Escherichia coli*. We found that HSR tends to adjust the folded conformation to reduce the harm of mutations, showing a high level of mutational robustness. Moreover, guanine preference in HSR was observed, supporting the hypothesis that the selective constraint for high structural stability may partly account for the high percentage of G content in *Escherichia coli* genome. Notably, we found a substantially reduced synonymous substitution rate in HSRs compared with that in their adjacent regions. Surprisingly and interestingly, the non-key sites in HSRs, which have slight effect on structural stability, have synonymous substitution rate equivalent to background regions. To explain this result, we identified compensatory mutations in HSRs based on structural stability, and found that a considerable number of synonymous mutations occur to restore the structural stability decreased heavily by the mutations on key sites. Overall, these results suggest a significant role of local structural stability as a selective force operating on mRNA, which furthers our understanding of the constraints imposed on protein-coding RNAs.

## Introduction

RNA molecules tend to adopt a folded conformation through the formation of Watson-Crick base pairing between complementary nucleotides. The resulting so-called RNA secondary structure emerges to be a key player in the regulations of gene expression [1]–[4]. By surveying secondary structures in various genomes, previous studies have revealed that a large number of genomes are being transcribed to produce non-coding RNAs that generally contain a conserved secondary structure [5]–[9]. The structural conformation of the molecule is often necessary for its functions. Precursor microRNAs (pre-miRNAs) are among the largest examples that illustrate the functions of secondary structure in non-coding RNAs. The pre-miRNA contains a ∼70-bp hairpin, which is recognized by the Dicer protein and then the loop region is removed to leave a dsRNA [10], [11]. The secondary structure in pre-miRNA is conserved during evolution [12]–[14], suggesting an important role of structural conformation in miRNA maturation. Interestingly, in recent years, a considerable number of protein-coding RNAs have been reported to contain local secondary structures [15]–[18]. Some translational processes, including translation initiation [19]–[21], co-translational folding of protein [22], [23], are sensitive to the variation of local structural stability. Moreover, a strong association between structural stability and protein abundance was observed in yeast [24]. These results suggest an important role of mRNA structural stability, which might be different from the roles of conserved secondary structures reported by previous studies. Besides its functions, numerous studies have focused on the evolution of RNA secondary structure [14], [25]–[29] and revealed several mechanisms to maintain the secondary structure, including lower substitution rate [30] and compensatory mutations [27].

Mutations that occur in the primary sequence might lead to a disruption of the paired regions, thus changing the *structural conformation* or *structural stability* of the molecule and impairing its original function. Various studies focused on the selective constraints in folded RNAs that are mostly located in non-coding regions [14], [30], [31]. They found a lower substitution rate in paired regions in comparison with that in unpaired regions [30]. Moreover, previous studies aimed at attributing the variation of GC content to the selection for high structural stability of RNA [32], [33]. The association has been observed in several types of non-coding RNA, such as miRNA. In miRNA, GC content is positively correlated with the organism's physiological temperature [33], suggesting a possible association between the base-pairing strength of miRNA-targets and the temperature of an organism. Unlike non-coding RNAs, multiple selective constraints, including structural stability [34], [35] and translation efficiency [36], [37], operate on mRNAs. Both two constraints influence the pattern of synonymous mutation. If there is selection on local protein-coding region for high structural stability, codons in such region might be under conflicting selective pressures: the codons promoting RNA folding with high structural stability might be translationally non-optimal. In this case, the locations of synonymous substitutions might be non-random with respect to the translational efficiency and structural stability. Knowledge of this conflict can further our understanding of constraints imposed on protein-coding RNAs. The initial studies of secondary structure, in mammals as well as yeast [38], [39], considered the thermodynamic stability of mRNA mediated by the changes in secondary structure, and revealed that C preference at the four-fold degenerate sites might be partly driven by the selection for RNA stability [38]. However, these studies did not focus on local protein-coding regions that form local secondary structures, exhibiting high structural stability. Numerous lines of evidence indicate that mRNA folding windows are small [40], [41]. Therefore, it is reasonable to investigate the substitution patterns of structural regions based on local folding instead of global folding. Moreover, the analysis of selective constraints on local structural stability of protein-coding region has not been performed in the genome wide scale. Therefore, the aim of this study is to analyze the natural selection related to the local structural stability from a genome wide perspective.

**Figure 1:**
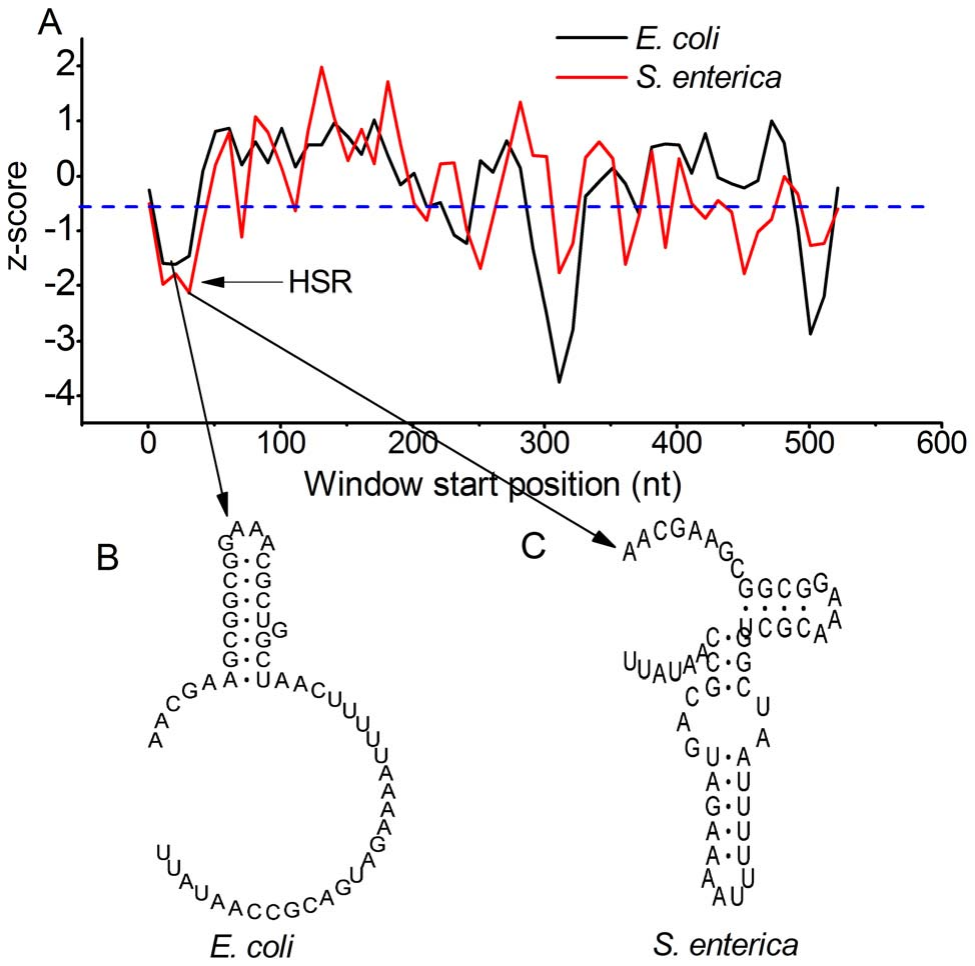
An example of HSRs in *Escherichia coli* and *Escherichia fergusonii*. A) Z-score is the normalized MFE. The threshold used to define HSR is marked by blue dashes. B) shows the secondary structures of HSRs. Although the HSRs between the two species are conserved (see Materials and Methods for details), the secondary structures are non-conserved.

In recent years, there has been a sharp growth in evidence showing widespread secondary structures in protein-coding region. In our previous study, we found that most of these structures exhibit high structural stability, while their structural conformations are non-conserved across different species [16]. We therefore identified the regions with high structural stability in *Escherichia coli* (HSR, high structural stability regions) using a loose threshold (Figure 1, Table S1-S2) [16], and revealed that number variation of HSR is correlated with gene functions, probably involving the regulations on the rhythm of translation elongation. However, the evolutionary pattern of HSR is still undetermined in that study. In particular, it remains unclear that how the structural stability of HSR is maintained under the constraint that multiple selective pressures are imposed on mRNA local regions. The selective pressure on HSR might be relaxed compared with that on structural RNA (e.g. 5 s rRNA), because there is a higher probability that a second mutation restores the structural stability disrupted by the first mutation. It is also of interesting to investigate the difference in the patterns of compensatory mutations between HSR and structural RNA. Therefore, in current study, we focus on the natural selections on HSRs, and aim at addressing the following questions that might advance our knowledge of selective pressures on mRNA: 1) Does selection for structural stability of HSR favor synonymous codons with high G/C? 2) How does HSR influence the local substitution rate of mRNA? 3) Since it is the structural stability rather than the structural conformation that is conserved, is the pattern of compensatory mutations in HSR different from that in structural RNA?

## Materials and Methods

### Coding sequence (CDS) and orthologs

Protein coding sequences of *Escherichia coli* K12 MG1655, *Escherichia fergusonii* ATCC and *Salmonella enterica* subsp. enterica serovar Typhi CT18 were downloaded from the National Center for Biotechnology Information FTP server (ftp://ftp.ncbi.nih.gov/genomes/). Sequences with length <200 nucleotides (nt) were excluded. In total, we obtained 4152, 4126 and 4246 coding sequences in *Escherichia coli*, *Escherichia fergusonii* and *Salmonella enterica*, respectively.

### Definition of HSR

HSR exhibits high structural stability, while not all HSRs have a conserved secondary structure. We thus identified HSRs on mRNAs only based on the minimum folding free energy (MFE) of local regions. The method was described previously [16]. The main steps are as follows. First, we calculated the normalized MFE, z-score, along CDS. For each CDS, we shuffled synonymous codons among sites with identical amino acids, controlling for amino-acid sequence, codon usage bias, and GC content. This process was repeated 100 times to generate 100 random sequences. We calculated MFE along CDS and the corresponding random sequences using a sliding window with 50 nt (approximately equal to the length of region (40 nt) covered by ribosome during elongation) in length and a step of 10 nt. MFE in each sliding window was calculated by RNAfold [42]. Z-score was calculated by equation (1):
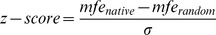
(1)where 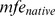 is the MFE of native sequence, 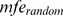 and 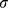 are the mean and standard deviation of MFE of 100 random sequences, respectively. Second, we used the following criteria to define HSR: 1) if a region contains more than two continuous sliding windows, in which the z-scores were all below the threshold of -0.65 [16], the region was defined as HSR. 2) If the percentage of the overlapping sites of two adjacent HSRs was higher than 50%, the two HSRs were combined.

### Mutational robustness of HSR

Mutational robustness of HSR refers to the sensitivity of structural stability to point mutations. We used two measures to estimate the mutational robustness of HSR. The first measure is the mean relative change of MFE over all single point mutations [35]. The second is the number of key sites. Key site indicates those sites, mutations on which result in more than 15% (other thresholds were also considered) increase in MFE. The two measures were obtained by performing the following analyses. First, for each site in HSR, three mutational sequences were generated by replacing original nucleotide with the other three nucleotides. Second, the MFE of the four sequences (one native and three mutational sequences) was calculated. The relative change of MFE was computed by equation (2):
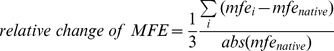
(2)where 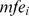 is the MFE of the i^th^ mutational sequence. 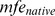 is the MFE of the native sequence. 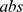 refers to the absolute value. Third, the relative changes at all sites were averaged and the number of key sites was computed.

As a control, for each HSR, we generated 30 random HSRs (rHSRs) by shuffling all synonymous codons with identical amino acid, maintaining amino acid sequence and codon usage. Moreover, the MFE of rHSR is similar to that of the corresponding HSR (located in MFE_HSR_±10% MFE_HSR_). The relative change of MFE and the number of key sites in random sequence are the average values of 30 random sequences.

### Calculation of local translation efficiency

We used tRNA adaptation index (tAI) [43] to measure the local translation efficiency. tAI was calculated by equation (3):
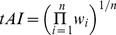
(3)where n is the length (codons) of HSR, w_i_ is the relative adaptiveness value of codon i, which was calculated according to the work of dos Reis et al. [43].

### Conserved HSRs and flank regions

**Figure 2:**
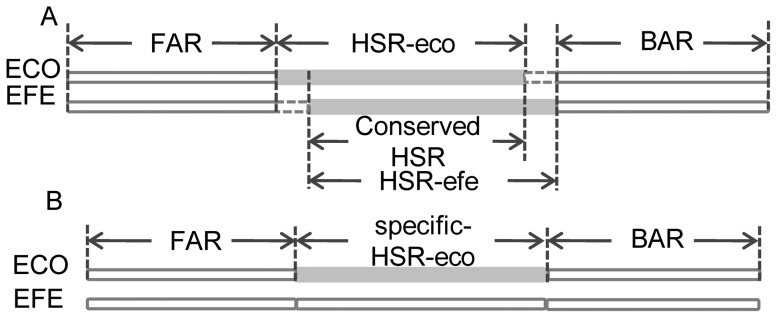
Definitions of conserved and specific HSRs. Conserved HSR is the overlapping region of the homologous HSRs between *Escherichia coli* and *Escherichia fergusonii* (HSR-eco and HSR-efe). ECO: *Escherichia coli*; EFE: *Escherichia fergusonii*; FAR (forward adjacent region) and BAR (backward adjacent region) refer to the two background regions, which have the same length to the corresponding HSR.

First, we obtained the orthologous relationship between *Escherichia coli* and *Escherichia fergusonii* from the KEGG database [44]. Only one-to-one orthologs were used in the analyses. In total, 3138 orthologs were extracted. Amino acid sequences of orthologs were aligned using MUSCLE [45]. The alignments were subsequently converted into mRNA sequence alignments. Considering that insertions and deletions (indels) strongly affect the positions of HSRs, we discarded the alignments with total indels >10 nt. 2676 alignments were left. Second, we defined the conserved HSRs between the two species. For each HSR in *Escherichia coli* (HSR-eco), we searched for the homologous HSR near the corresponding region of orthologs in *Escherichia fergusonii* (HSR-efe). If HSR-efe was found and the percentage of overlapping sites between HSR-eco and HSR-efe was higher than 50%, the overlapping regions of the two HSRs were defined as conserved HSRs (Figure 2A). The other HSRs existing in only one species were defined as specific HSRs (Figure 2B). Two background regions: FAR and BAR (FAR: forward adjacent region, BAR: backward adjacent region) were extracted and used as controls. The three regions have the same length. Considering that the substitution rate and GC content in the first 200 nt are significantly different from that in other regions (Figure S1), we discarded the data set FAR-HSR-BAR if FAR is located in the first 200 nt of CDS.

### Estimation of substitution rate

For each orthologs, all sub-alignments covered by conserved HSRs were concatenated. The concatenated alignments with length <100 nt were excluded. In total, 1217 alignments were remained. The concatenated alignments of FAR and BAR were obtained using the same method. The estimation for synonymous substitution rate (dS) was performed using the CODEML program of the PAML package [46] with runmode -2. Alignments with dS >3 were excluded.

### Identification of compensatory mutations

We only considered single point mutations to investigate the pattern of compensatory mutations occurring in HSRs. First, we reconstructed the ancestral sequences of *Escherichia coli* and *Escherichia fergusonii* using the maximum likelihood method [47]. Alignments with idels >10 nt were not included. The indels in ancestral sequences were inferred by parsimony method using *Salmonella enterica* as outgroup. Again, HSRs in ancestral sequences and the conserved HSRs between ancestral and extant sequences, termed ancestral-extant HSRs, were defined using the same method as described above. Ancestral HSRs containing indels were discarded. In total, 11583 pairs of ancestral-extant HSRs were obtained.

**Figure 3:**
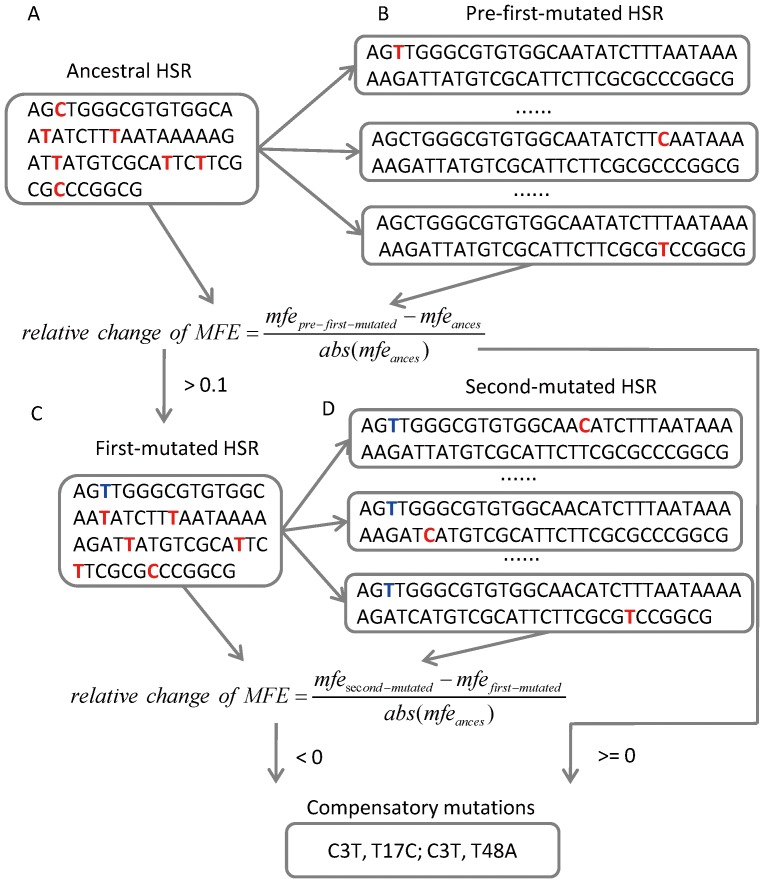
Overview of the method to identify the compensatory mutations. A-B: we generated the pre-first-mutated HSR by introducing one mutation to ancestral HSR based on the synonymous mutations in ancestral-extant HSRs. The mutated site in pre-first-mutated HSR is marked in red. First mutations were defined as the mutations that increase the MFE of ancestral-HSR, the corresponding pre-first-mutated was termed first-mutated HSR. C-D: second-mutated HSR was generated by introducing one mutation to the first-mutated HSR. First mutation is marked in blue. The mutated site in second-mutated HSR is marked in red.

Second, we defined the *first mutations* in ancestral-extant HSRs. For this purpose, we generated the pre-first-mutated HSR by introducing one mutation to ancestral HSR based on the synonymous substitutions in ancestral-extant HSRs (Figure 3A-3B). The relative change of MFE between pre-first-mutated and ancestral HSRs was calculated by equation (4):
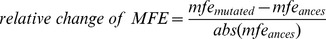
(4)where 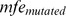 is the MFE of mutated HSR. 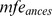 refers to the MFE of ancestral HSR. 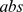 refers to the absolute value. Mutations with relative change >10% were defined as the first mutations, and the corresponding mutated HSRs were termed *first-mutated* HSRs.

Third, we identified the *second mutations* occurring in the first-mutated HSRs using the same method (Figure 3C-3D). The second mutation refers to the mutation that decreases the MFE of the first-mutated HSR (i.e. the relative change of MFE between the first-mutated and second-mutated HSR <0, Figure 3). Moreover, the second mutations, which decrease MFE of ancestral HSR without the occurrence of the first mutation (in this case, the second-mutated HSR can be treated as the pre-first mutated HSR), were excluded. The left second mutations were defined as the *compensatory mutations* corresponding to the first mutation (Figure 3).

As a control, for each pair of ancestral-extant HSRs, we simulated random extant HSR by randomly generating the synonymous mutations on ancestral HSR based on the number of synonymous substitutions in ancestral-extant HSRs and the genomic frequency of codons in extant species. This process was repeated 30 times to generate 30 pairs of ancestral-random HSRs. The compensatory mutations in ancestral-random HSRs were defined using the same method as described above.

## Results

### High mutational robustness of HSR

Mutational robustness is the ability of genotypes to display high tolerance against mutations, which is considered a fundamental feature of biological systems, from single molecules to gene regulatory networks [48], [49]. To maintain its functions, HSR is assumed to evolve to keep a high level of mutational robustness. Table 1 summaries the two measures to estimate the mutational robustness. In total, we identified 6352 conserved HSRs between *Escherichia coli* and *Escherichia fergusonii* (Table 1, Table S3), which are located in 2256 genes (84.3% of all orthologs). In addition, 4202 and 4306 specific HSRs were detected in *Escherichia coli* and *Escherichia fergusonii*, respectively. We found that both conserved and specific HSRs have lower mean relative changes of MFE over all point mutations on HSRs (paired *t*-test, all *p*-values <10^−16^, Table 1), compared with the corresponding random HSRs with the same features. Considering that the difference in MFE between the native and random HSR (the MFE of random HSR is similar, not equal, to that of the native HSR) might affect the relative change of MFE, we also calculated the mean absolute change. The difference remains significant (paired *t*-test, all *p-*values <10^−16^, Table 1). The results suggest that HSRs have high tolerance to point mutations. Moreover, we computed the number of key sites, which strongly decrease the structural stability of HSR. Again, we found a lower number of key sites in both conserved and specific HSRs in comparison with that in random HSRs (paired *t*-test, all *p*-values <10^−16^, Table 1, Figure S2), indicating that there is a tendency to adjust the folded conformation of HSR to reduce the number of the sites, which have significant effect on structural stability. Overall, these results indicate that HSRs evolve to maintain high mutational robustness.

**Table 1:** Mutational robustness of HSRs.

HSR classification	Number of HSRs	Absolute change of MFE (95% CI b)	Relative change of MFE (95% CI)	Number of key sites (95% CI)
Conserved HSR	6352	0.423 (0.409, 0.437)	0.0148 (0.0143, 0.0154)	3.337 (3.273, 3.460)
Conserved rHSR a	6352	0.501 (0.493, 0.509)	0.0188 (0.0185, 0.0191)	3.940 (3.875, 4.006)
HSR-eco	4202	0.367 (0.351, 0.384)	0.0137 (0.0130, 0.0144)	3.501 (3.391, 3.612)
rHSR-eco	4202	0.439 (0.430, 0.449)	0.0187 (0.0184, 0.0190)	4.169 (4.094, 4.243)
HSR-efe	4362	0.368 (0.352, 0.385)	0.0138 (0.0131, 0.0146)	3.778 (3.664, 3.893)
rHSR-efe	4362	0.435 (0.425, 0.444)	0.0176 (0.0172, 0.0180)	4.357 (4.284, 4.430)

a rHSR: random HSR, which was generated by shuffling synonymous codons among sites with identical amino acids, while maintaining amino acid sequence, codon usage bias, and GC content. In addition, the MFE of rHSR is similar (located in MFE_HSR_±10%) to that of native HSR. HSR-eco (HSR-efe): the HSRs only exist in *Escherichia coli* (*Escherichia fergusonii*).

b CI: confidence interval.

### Guanine preference at the third sites

Although high percentage of GC content promotes RNA folding with high structural stability because GC pairs are more stable than AT pairs, it is not obvious that HSRs maintain high structural stability by increasing G/C content during evolution. There are multiple selective pressures, including selections for RNA folding and translation efficiency, appearing to affect the patterns of synonymous mutation. Therefore, increasing GC content does not always benefit translational regulation. In particular, codon order in HSR instead of base composition might be adjusted to meet the requirements for structural stability and translation efficiency.

**Figure 4:**
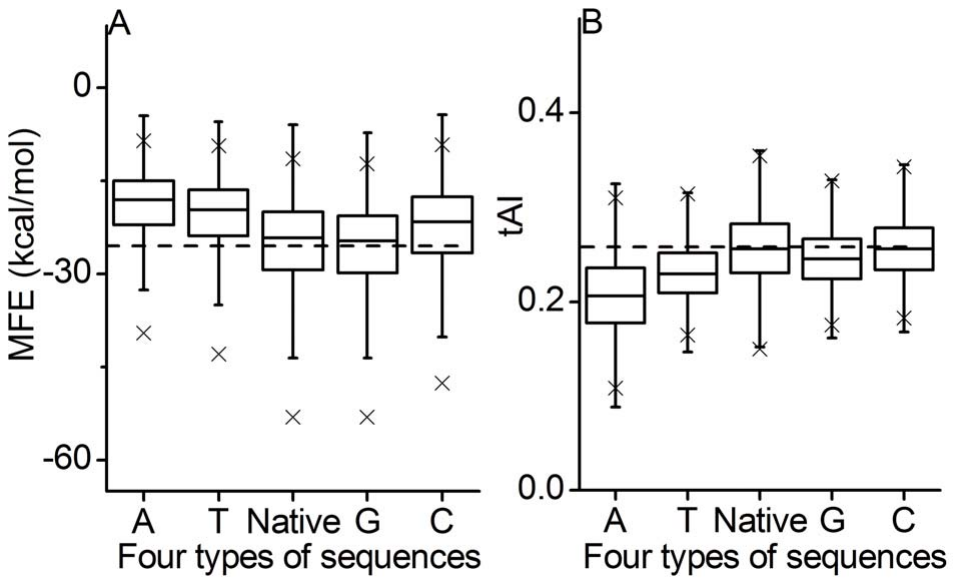
Effect of base composition on MFE and tAI. Four types of sequences refer to the substituted HSRs replacing nucleotides at the four-fold degenerate sites with A, T, G, or C, respectively. The mean MFE (A) and mean tAI (B) of native HSRs are indicated by dashes.

Here, we asked whether GC content in HSR is under selection for high structural stability. We analyzed GC content at the four-fold degenerate sites in the three regions: FAR, HSR and BAR. We found that HSR exhibits significantly higher G content than the other two regions (paired *t*-test, all *p*-values <10^−16^, Table 2). There is also a slightly increasing C content in HSRs (paired *t*-test, *p*-values <0.01). These results indicate a selection for increasing G and C (especially for G) in HSRs. Meanwhile, by checking local translation efficiency measured by tAI, we found a trend towards increasing translation efficiency in HSRs (paired *t*-test, *p*-values <10^−5^, Table 2). Therefore, the possibility remains that the selection for translation efficiency might be responsible for the high G/C content observed in HSRs. To resolve this issue, we simulated random mutations on HSRs by replacing the nucleotides at the four-fold degenerate sites with other nucleotides. For each HSR, half of the four-fold degenerate sites were randomly selected and mutated to generate four types of substituted sequences: G-Seq (G rich sequence, replacing nucleotides with G, the same to others), C-Seq, T-Seq, and A-Seq. To exclude the effect of substituted positions, for each type of sequence, we generated 50 substituted sequences, and calculated the mean MFE difference between native and substituted sequences. We found that G-seqs have a lower mean MFE than native sequences (paired *t*-test, *p*-value <10^−16^), whereas other three types of substituted sequences show significantly increased MFE (paired *t*-test, all *p*-values <10^−16^, Figure 4A), suggesting that increasing A/T/C (especially for A/T) or decreasing G in HSR will decrease the structural stability of HSR. Interestingly, we found an opposite pattern when comparing local translation efficiency between substituted and native sequences. Only C-seqs have a higher mean value of tAI, which is approximately equal to that of native sequences (0.257 in C-seqs vs. 0.255 in native sequences). The other three types of substitutions significantly decrease the translation efficiency in HSRs (paired *t*-test, all *p*-values <10^−16^, Figure 4B). In addition, considering that HSRs are G preference, G-seqs have a lower number of substitutions than the other types of sequences, which might influence the significance inferred from G-seqs, we thus performed a similar analysis using the HSRs with G content <0.25. Similar results were obtained (Figure S3). Overall, these results suggest that the increased G content in HSRs results from the selection for maintaining high structural stability.

**Table 2:** Comparison of base composition among three regions.

Species	Regions	MFE (kcal/mol)	Base Composition				tAI
			A	T	G	C	
ECO	FAR	−22.25 (7.55)	0.131 (0.072 a)	0.231 (0.092)	0.355 (0.105)	0.284 (0.093)	0.250 (0.033)
	HSR	−25.21 (7.73)	0.107 (0.068)	0.195 (0.088)	0.412 (0.106)	0.285 (0.088)	0.255 (0.033)
	BAR	−22.23 (7.47)	0.136 (0.075)	0.229 (0.096)	0.359 (0.108)	0.277 (0.089)	0.249 (0.034)
EFE	FAR	−21.57 (7.13)	0.151 (0.076)	0.252 (0.095)	0.333 (0.104)	0.264 (0.088)	0.282 (0.039)
	HSR	−24.73 (7.68)	0.130 (0.074)	0.213 (0.088)	0.385 (0.108)	0.271 (0.084)	0.286 (0.039)
	BAR	−20.94 (7.08)	0.155 (0.081)	0.249 (0.092)	0.332 (0.104)	0.263 (0.085)	0.280 (0.039)

a The standard deviations are shown in parentheses.

### Substitution rate variation

**Figure 5:**
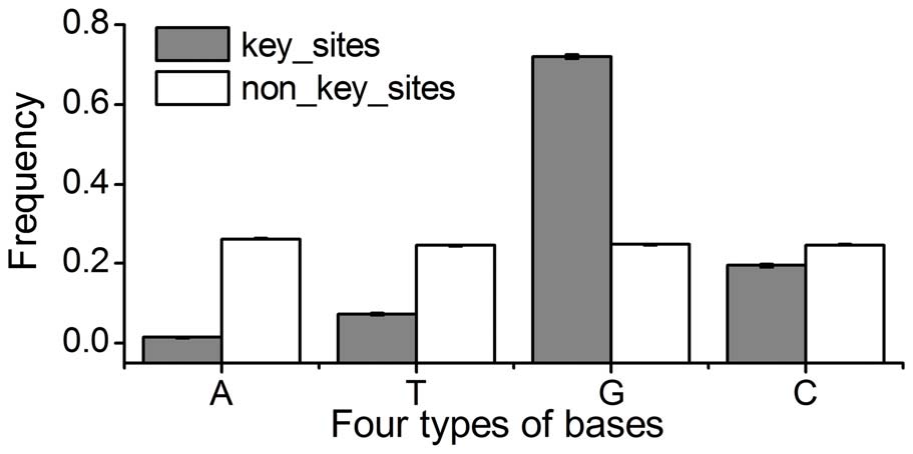
Comparison of base composition between key and non-key sites. Key sites indicate those sites, mutations on which result in >15% increase in MFE.

**Figure 6:**
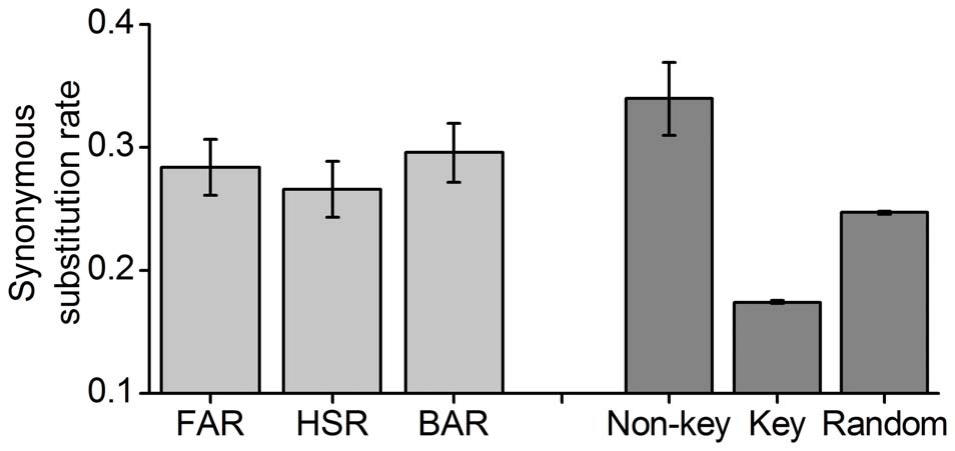
Comparison of synonymous substitution rates among three regions. FAR: forward adjacent region; BAR: backward adjacent region. Random indicates the random codons, which are identical to the codons of key sites while are located in other regions. The 95% confidence intervals of synonymous substitution rates of key and non-key sites were estimated by bootstrap methods.

Mutational robustness is correlated with structural functionality and complexity. The results in previous sections showed that the number of key sites in HSR is lower than that in the corresponding random HSR. In addition, we compared base compositions of key sites and non-key sites, and found that key sites are G preference. About 70.2% key sites are G, while the percentage in non-key sites is about 24.7% (Figure 5). Note that HSRs with high percentage of G are sensitive to mutation, making it difficult to maintain high mutational robustness. In this case, one might expect lower dS in HSRs to reduce the harm of mutations. Indeed, we found a reduced dS in HSRs compared with that in the other two regions (Wilcoxon test, all *p*-values <0.05, Figure 6). Moreover, we estimated the dS of the codons that contain key sites. Again, the dS is significantly lower than that of the other codons in HSR (Wilcoxon test, *p*-value <10^−5^, Figure 6). Considering that the codons with key sites are G preference, which might affect the inferred substitution pattern, we randomly extracted the codons in other regions (non-HSRs), which are identical to the codons with key sites in HSRs. We estimated the dS of these “random” codons, and found a higher dS compared with that of the codons with key sites (Wilcoxon test, *p*-value <10^−5^). Overall, the results that HSRs have lower synonymous substitution rate, especially for the codons with key sites, suggest a selective constraint imposed on HSRs to reduce the harm of mutations.

### Compensatory mutations in HSRs

Distinct positions in structural RNA may not evolve independently because of shared structural or functional constraints. Considering the RNA with functional secondary structure, mutations on some sites (key sites) might disrupt the secondary structure, thus disabling its original function. Besides the strategy involving the reduction of the substitution rate on key sites (as suggested in previous section), another strategy is used to decrease the harm of the mutations on key sites. That is, a second (so called “compensatory”) mutation occurs on the specific site to restore the original conformation [27], [50], [51]. Previous studies on conserved secondary structure have revealed that the compensatory mutations should have a fitness equivalent to the wild type, resulting in an increasing of the substitution rate among these specific sites compared with that of key sites [52].

By analogy with the conserved secondary structure, compensatory mutations might be observed in HSRs, although it is the structural stability instead of the structure conformation that is maintained during evolution. Indeed, we found that the dS of non-key sites is higher slightly than that of background regions (0.339 in non-key sites vs. 0.283 in FAR and 0.295 in BAR, Figure 6), suggesting a relaxed selective constraint on non-key sites in HSRs compared with that on key sites. Consequently, we asked whether the compensatory mutations occurring on non-key sites account for this result. We first compared the effect of observed synonymous mutations on MFE between the ancestral-extant and ancestral-random HSRs. We found that the mean relative change of MFE caused by synonymous mutations in ancestral-extant HSRs is significantly lower than that in ancestral-random HSRs (0.028 vs. 0.060, paired *t*-test, *p*-value <10^−16^), indicating that the locations and patterns of synonymous mutations are non-random with respect to structural stability. This result also indicates a possibility that compensatory mutations occur to decrease the overall relative changes of MFE caused by mutations.

We then identified the compensatory mutations for each synonymous mutation that strongly increases the MFE of HSR. In two species, 2294 synonymous mutations (9.60% of all synonymous mutations) were extracted and treated as the first mutations (Table 3). In 41.70% of the first mutations in *Escherichia coli*, compensatory mutations were observed. The percentage is higher (*t*-test, *p*-value <10^−16^, Table 3) than that in ancestral-random HSRs. A similar pattern was observed in *Escherichia fergusonii* (Table 3). Note that only single point mutations were used and the conjugated effect of two or more than two point mutations was not considered. The true percentage of the compensatory mutations could be substantially higher than the observed value.

**Table 3:** Summary of compensatory mutations.

Type	Species	Number of first mutations	Number of compensatory mutations	Percentage c (%)	Paired/Unpaired d
I^a^	ECO b	1115	691	41.70	0.578
	Random	1693.3 (34.6)	637.0 (28.8)	28.59 (1.08)	0.657 (0.045)
	EFE	1719	842	33.92	0.520
	Random	1668.8 (24.2)	639.7 (31.7)	28.77 (1.19)	0.614 (0.050)
II	ECO	931	337	9.47	–
	Random	607.0 (25.3)	823.5 (30.8)	67.6 (1.96)	–
	EFE	778	364	9.35	–
	Random	695.8 (15.9)	950.2 (39.4)	67.9 (1.66)	–

a Type refers to the two types of first mutations. I indicates the first mutations, which increase the MFE of ancestral HSR; II indicates the first mutations, which decrease the MFE of ancestral HSR (see text for details).

b ECO (EFE) refers to the pair of ancestral-extant HSRs in *Escherichia coli* (*Escherichia fergusonii*); Random refers to the pair of ancestral-random HSRs, which were obtained by randomly generating the synonymous mutations based on the number of synonymous substitutions in corresponding ancestral-extant HSRs and the genomic frequency of codons.

c Percentage refers to the percentage of the first mutations, which decrease the MFE of ancestral HSR while compensatory mutations exist to partly restore this disruption. Two or more compensatory mutations were observed in a few first mutations. Thus, the percentage is lower than the ratio: Number of compensatory mutations/Number of first mutations.

d Paired means that the first mutated base is paired with the second mutated base, while unpaired means that the two mutated bases are unpaired.

We classified all compensatory mutations into two types: paired and unpaired. Paired means that the first mutated base is paired with the second mutated base, while the unpaired means that the two mutated bases are unpaired. We calculated the ratio paired/unpaired and surprisingly found that the ratio is lower than the expected value in the two species (*t*-test, all *p-*values <10^−7^, Table 3), which is different from the pattern inferred from the conserved secondary structure (the ratio is higher than the expected value) [27]. This result is consistent with the idea that structural stability instead of structural conformation in HSR is conserved during evolution [16], [35].

In addition, we redefined the first mutations as the synonymous mutations that result in >10% **decrease** in MFE of ancestral HSR. We performed a similar analysis as above based on the new first-mutated HSRs to test whether there are compensatory mutations that restore the MFE decreased by the first mutation. Interestingly, compensatory mutations were found only in 9.47% of the first-mutated HSRs in *Escherichia coli*, which is significantly lower (*t*-test, *p-*value <10^−16^, Table 3) than that in random mutations. A similar pattern was found in *Escherichia fergusonii* (Table 3). The results suggest that only a few mutations that increase structural stability of HSR are subject to negative selection. Overall, the findings suggest that a compensatory mechanism exists to maintain the high structural stability of HSRs.

## Discussion

mRNA is a key component of a complex regulatory network. It accommodates numerous regulatory signals delineated along the protein coding regions in an intricate overlapping manner [53]. A worthy issue is that how these signals evolve to meet the requirements of the regulations on different levels of translation, and how the evolutionary patterns of these regulatory elements affect the observed evolutionary pathway of genome. In this study, we focused on one of the most important regulatory elements, mRNA secondary structure, and investigated their evolutionary patterns. HSR is a special region on mRNA containing a local secondary structure. A considerable proportion of mRNAs are covered by HSRs (about 30% on average). Therefore, base compositions and substitution rate of mRNA might be remarkably affected by HSRs.

In current study, we found that HSRs have high mutational robustness compared with random HSRs. It suggests that the folded conformation of HSR is adjusted to reduce the harm of mutation. We subsequently asked how the mutational robustness is maintained. Since base composition has strong effect on structural stability of HSR, we thus focused on the GC content of HSR. The results showed that HSRs are G preference, supporting the hypothesis that the selective constraint for high structural stability might partly account for high percentage of G in *Escherichia coli* genome. Note that the observed G contents in the other two background regions are higher than the other three bases. This suggests that there are other selective pressures imposed on mRNA, resulting in the variation of G content among sites, as suggested by the previous studies [4], [34], [54].

Our result is different from the claim in the work of Chamary et al. [38], which showed that mRNA stability partly drives C preference in *Mus musculus*. To explain the difference, we analyzed the relationship between the local stability and base composition of the 70 coding sequences used in their study. Again, we found that G content at the four-fold degenerate sites in HSRs is higher than that in background regions (0.276 in HSR vs. 0.238 in FAR, Wilcoxon test, *p-*value  = 0.050, and 0.227 in BAR, *p-*value  = 0.011, ). Although there is a universal excess of C over G, the difference in C contents among FAR, HSR and BAR is not significant (Wilcoxon test, all *p*-values values >0.1, Figure S4). In addition, we compared the mean MFE of the four types of substituted sequences, and found that the pattern is similar with that in *Escherichia coli* (Figure S5). Note that global mRNA was folded in the work of Chamary et al. [38], which might be involved in the global regulation of translation, such as RNA decay. We focused on local structural stability, which regulates a series of co-translational processes. Therefore, the difference between the two studies might result from the different methods dealing with RNA folding.

Although high percentage of G promotes HSR folding with high stability, increasing G will decrease the mutational robustness of HSR. An efficient strategy is to keep low substitution rate in HSR, especially for the key sites. As expected, a lower dS in HSR was observed. This result is consistent with the findings in a recent work [55], which revealed a significant correlation between mRNA structural stability and synonymous rate, as well as structural stability and non-synonymous substitution rate [55]. Moreover, note that horizontal gene transfer (HGT) occurs frequently in *Escherichia coli* [56]. The results might be affected by HGT events. Therefore, we discarded the predicted HGT genes, obtained from the works of Garcia-Vallvé et al. [57], and re-estimated substitution rate and GC content in HSRs. Similar results were obtained (Figure S6).

**Figure 7:**
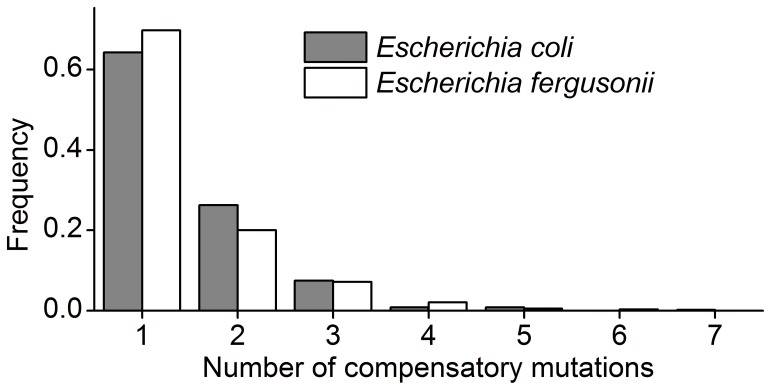
Distribution of the number of compensatory mutations in HSR. In more than 30% of the first-mutated HSR with compensatory mutations, two or more compensatory mutations were detected.

The dS in non-key sites is approximately equal to that in background regions. We proposed that compensatory mutations, occurring in about 40% first-mutated HSRs, partly account for this result (Table 3). Moreover, we calculated the number of compensatory mutations, and found that two or more compensatory mutations were detected in more than 30% first-mutated HSRs (Figure 7). This result suggests a different type of compensatory evolution compared with that occurring in the structural RNAs, in which the compensatory mutation is site-specific, and co-evolution would be observed during evolution. In HSRs, however, the substitution patterns of a large number of sites are affected by key sites, making it difficult to detect an obvious co-evolution. In addition, we only considered single point mutations to detect the compensatory mutations. In fact, it is more likely that multiple mutations coordinate the folded conformation to restore the disrupted MFE. The conjugated effect of multiple mutations are worth pursuing at a deeper level.

## Supporting Information

Figure S1 **GC content and sequence identity along mRNA.** In the first 30 codons of mRNA, GC content (A) at the three positions of codon is significantly lower than that in other regions. The sequence identity (B) in the first 50 codons is different from the latter regions.
(TIF)

Figure S2 **Number of key sites under different thresholds.** The number of key sites in native HSRs is significantly lower (paired *t*-test, all *p*-values <10^−16^) than that in random HSRs when threshold <0.2. In both native and random HSRs, the numbers of key sites are close to 0 when threshold >0.2.
(TIF)

Figure S3 **Comparison of MFE and tAI among four types of substituted sequences.** Four types of sequences refer to the substituted HSRs replacing nucleotides at the four-fold degenerate sites with A, T, G, or C, respectively. The mean MFE (A) and tAI (B) of native HSRs is indicated by dashes, respectively. The data was based on the HSRs with G<0.25.
(TIF)

Figure S4 **Comparison of base composition in three regions, showing G preference in HSRs.** The data were obtained based on 70 mRNAs in *Mus musculus*.
(TIF)

Figure S5 **Comparison of MFE and tAI among four types of substituted sequences.** The data were calculated based on 70 mRNAs in *Mus musculus*.
(TIF)

Figure S6 **Comparisons of synonymous substitution rates and base compositions in the three regions.** The data were obtained by excluding the genes detecting the horizontal gene transfer event.
(TIF)

Table S1 **Positions of HSRs in** ***Escherichia coli*** **.**
(XLS)

Table S2 **Positions of HSRs in Escherichia fergusonii.**
(XLS)

Table S3 **Positions of conserved HSRs between** ***Escherichia coli*** **and** ***Escherichia fergusonii*** **.**
(XLS)
