# Supplementary figures and images for: Genome-Wide Analysis of Selective Constraints on High Stability Regions of mRNA Reveals Multiple Compensatory Mutations in Escherichia coli
Source: PLoS One. 2013 Sep 27;8(9):e73299. doi: 10.1371/journal.pone.0073299 (PMC3785496; doi:10.1371/journal.pone.0073299)

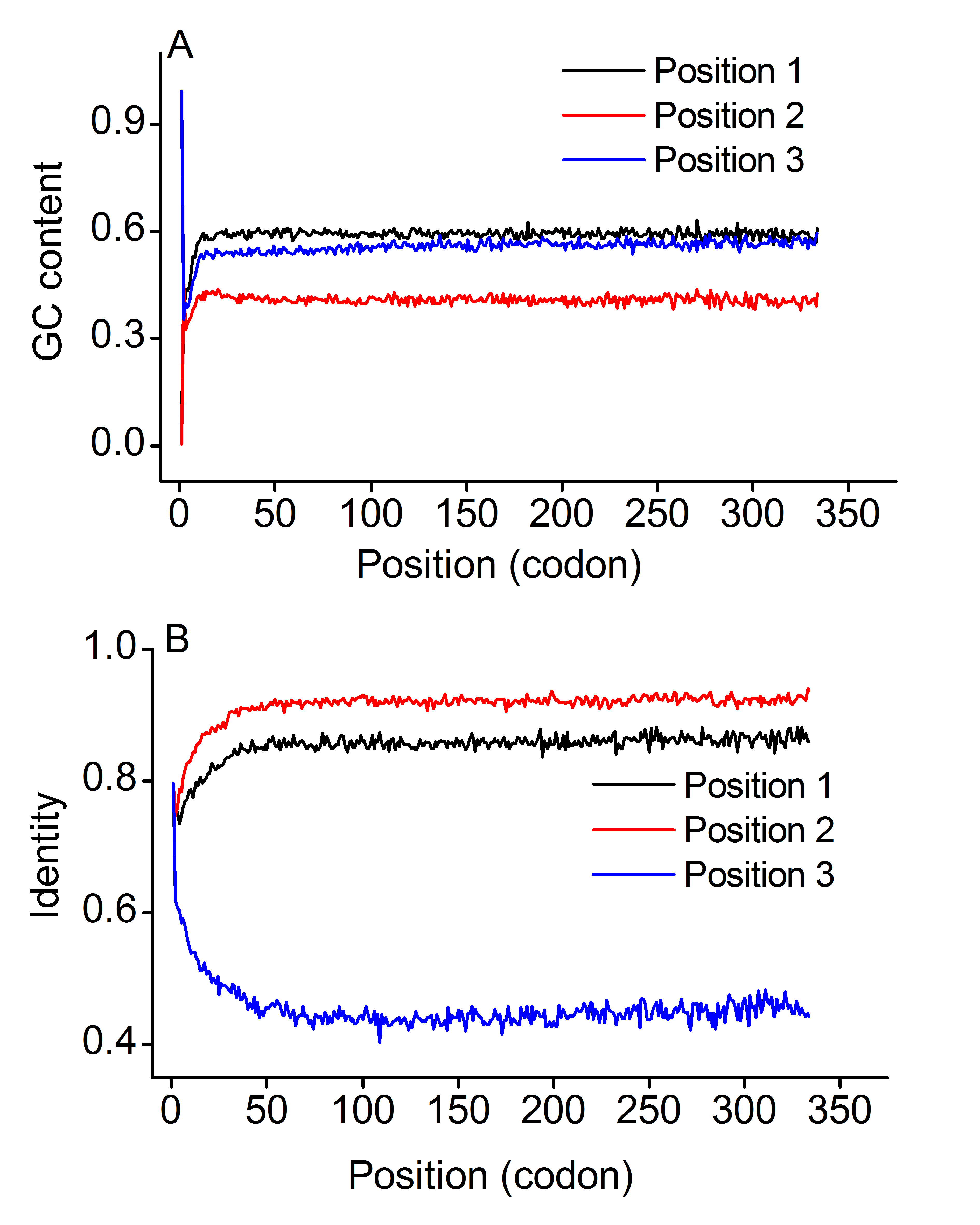

Supplement: Figure S1 — GC content and sequence identity along mRNA. In the first 30 codons of mRNA, GC content (A) at the three positions of codon is significantly lower than that in other regions. The sequence identity (B) in the first 50 codons is different from the latter regions. (TIF) [file pone.0073299.s001.tif]

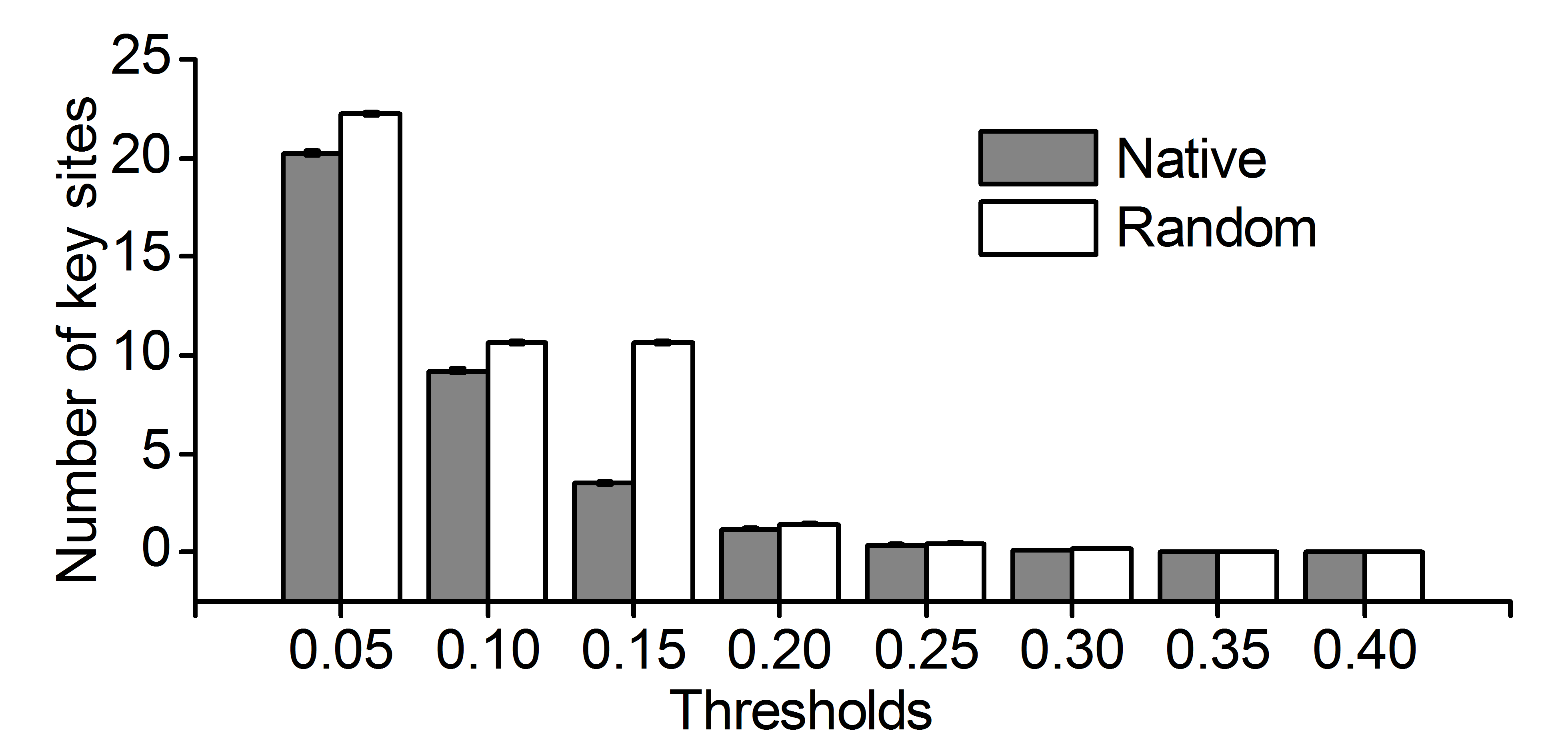

Supplement: Figure S2 — Number of key sites under different thresholds. The number of key sites in native HSRs is significantly lower (paired t-test, all p-values <10−16) than that in random HSRs when threshold <0.2. In both native and random HSRs, the numbers of key sites are close to 0 when threshold >0.2. (TIF) [file pone.0073299.s002.tif]

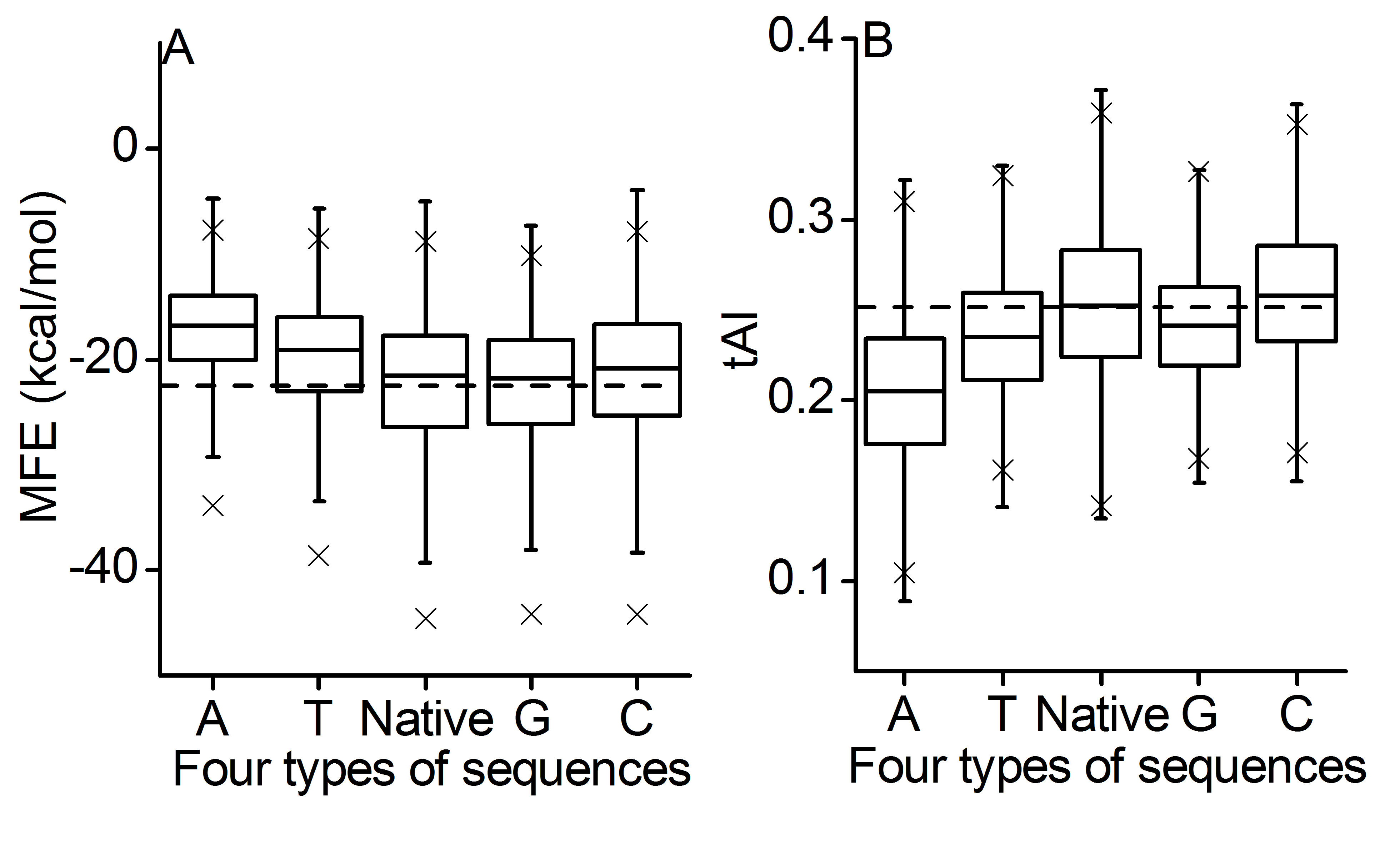

Supplement: Figure S3 — Comparison of MFE and tAI among four types of substituted sequences. Four types of sequences refer to the substituted HSRs replacing nucleotides at the four-fold degenerate sites with A, T, G, or C, respectively. The mean MFE (A) and tAI (B) of native HSRs is indicated by dashes, respectively. The data was based on the HSRs with G<0.25. (TIF) [file pone.0073299.s003.tif]

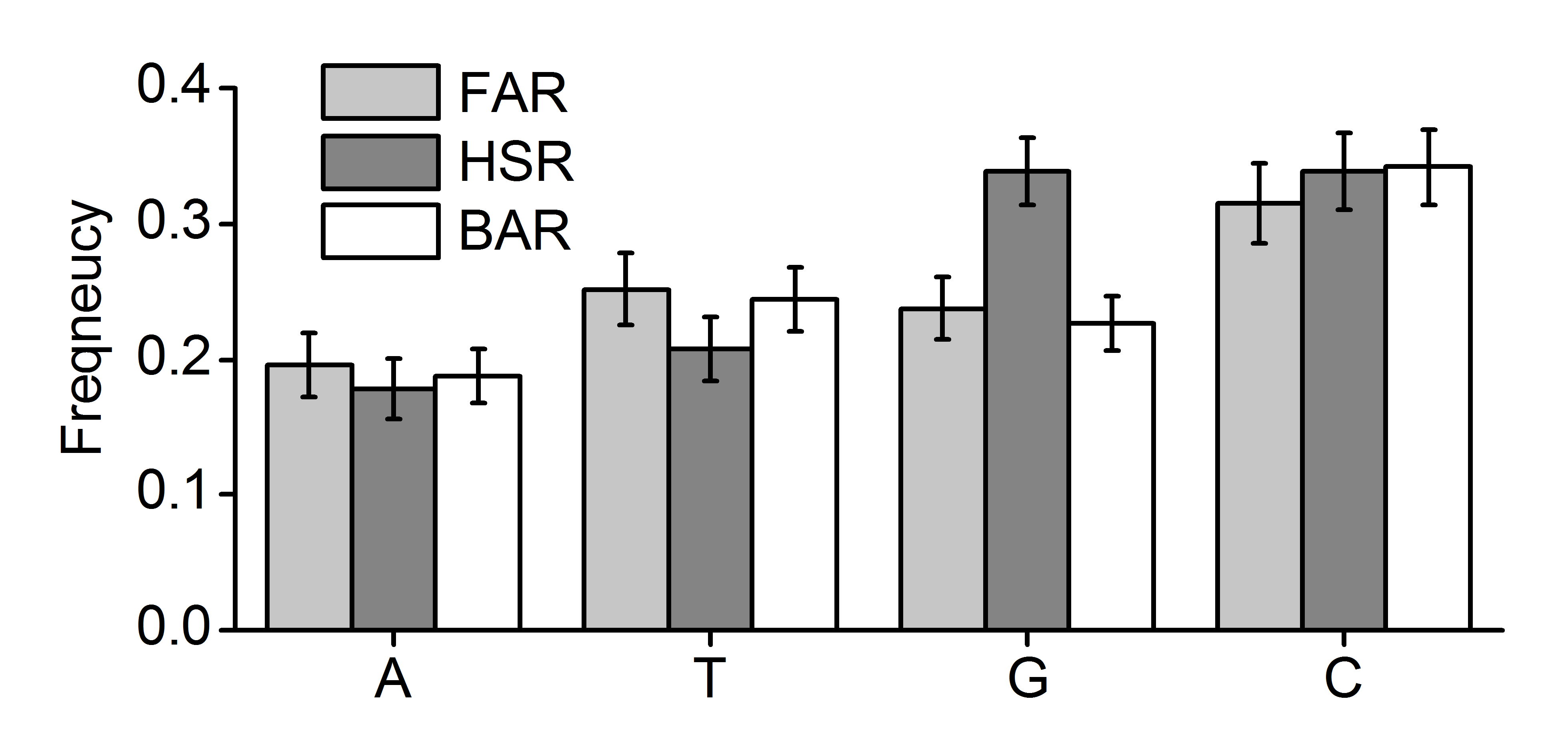

Supplement: Figure S4 — Comparison of base composition in three regions, showing G preference in HSRs. The data were obtained based on 70 mRNAs in Mus musculus. (TIF) [file pone.0073299.s004.tif]

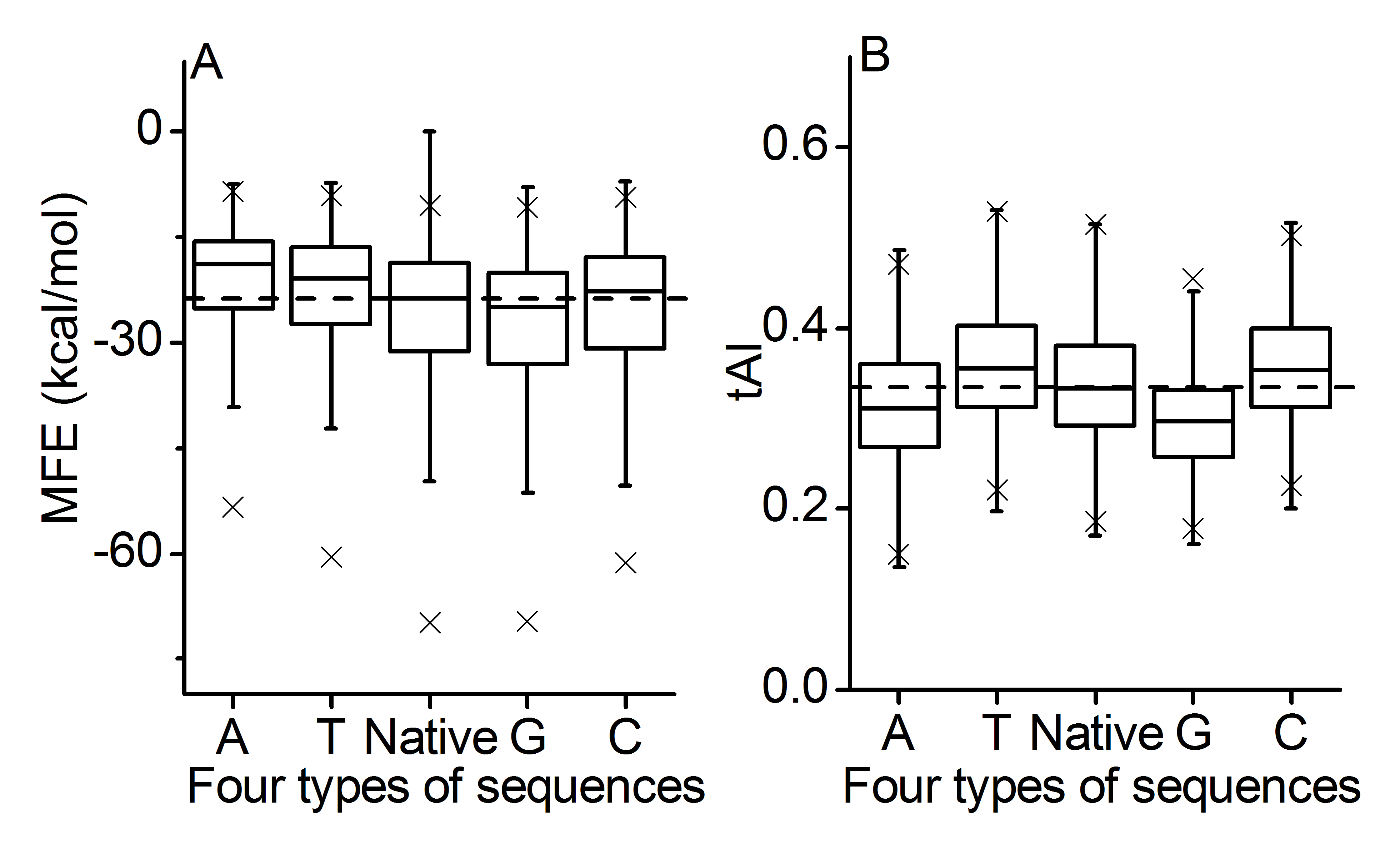

Supplement: Figure S5 — Comparison of MFE and tAI among four types of substituted sequences. The data were calculated based on 70 mRNAs in Mus musculus. (TIF) [file pone.0073299.s005.tif]

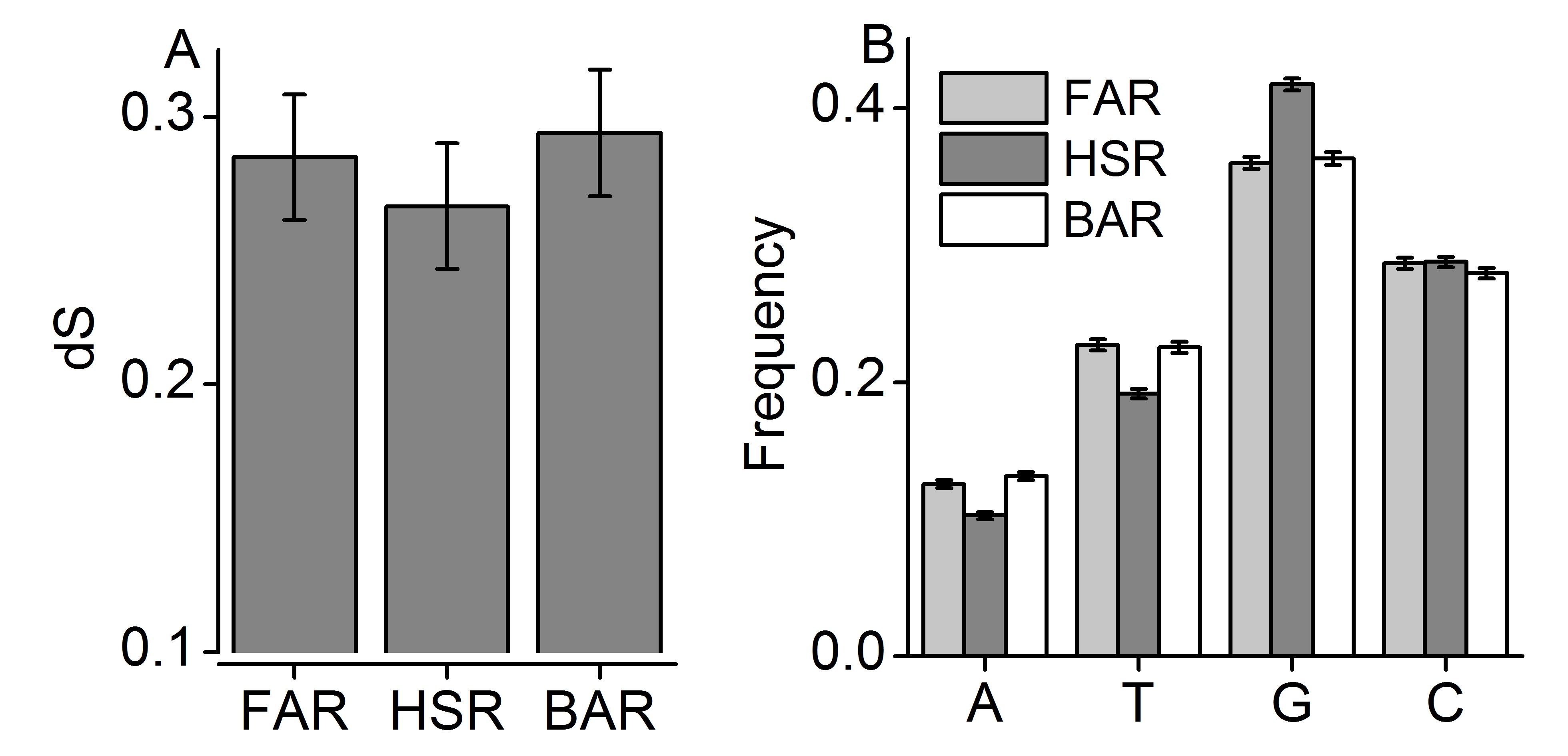

Supplement: Figure S6 — Comparisons of synonymous substitution rates and base compositions in the three regions. The data were obtained by excluding the genes detecting the horizontal gene transfer event. (TIF) [file pone.0073299.s006.tif]
